# The Defense Mechanism of *PpCAD4* in *Physcomitrium patens* Against *Botrytis cinerea*

**DOI:** 10.3390/plants15030413

**Published:** 2026-01-29

**Authors:** Mao Wu, Guiqing Li, Xiaoai Wu, Huan Zhao, Mei Li, Yanan Hu, Shan Jiang, Huiqing Yan

**Affiliations:** 1 School of Life Sciences, Guizhou Normal University, Guiyang 550001, China; 232100100410@gznu.edu.cn (M.W.);; 2 College of International Education, Guizhou Normal University, Guiyang 550001, China

**Keywords:** *Physcomitrium patens*, cinnamyl alcohol dehydrogenase, *Botrytis cinerea*, flavonoid, defense response

## Abstract

The existence of lignin in *Physcomitrium patens* has been controversial. However, cinnamyl alcohol dehydrogenase (CAD), the key enzyme in monolignol biosynthesis, has been identified with four gene members in *P. patens*. Despite the roles of *PpCAD1* in moss architecture being proven in a previous study, the functions and molecular mechanisms of *PpCAD4* remain largely unexplored in early terrestrial plants. This study aims to unravel this mystery via a comprehensive analysis of the transcriptome and metabolome of *PpCAD4*-overexpression (OE) lines compared with wild type (WT) under *Botrytis cinerea* treatment, firstly. A total of 475 and 1368 significantly differentially expressed genes in *PpCAD4*-OE lines compared to the wild type at 6 h and 12 h post-inoculation, which were predominantly enriched in pathways involving flavonoid, phenylpropanoid biosynthesis, and plant hormone signal transduction. Concurrently, metabolomic profiling revealed 160 and 114 differentially accumulated metabolites in *PpCAD4*-OE at the corresponding time points, with phenolic acids and flavonoids collectively constituting over 45% of these compounds. Furthermore, the MADS-box transcriptional factor PpMC6 negatively regulated *PpCAD4* expression by yeast-one-hybrid and dual-luciferase assays. Finally, Catalase isozyme 2 (PpCAT2) and E3 ubiquitin-protein ligase (PpE3) were identified as interactive partners with PpCAD4, respectively, deducing that the increasing of reactive oxygen species might be promoted by PpCAT2 degradation through PpE3 after *B. cinerea* assault. Our results demonstrated that the essential roles and potential mechanisms of *PpCAD4* are essential for defense against pathogens during the adaptation to land in moss.

## 1. Introduction

The plant transition from aquatic to terrestrial adaptation is largely due to the emergence and evolution of a large group of so-called “phenolic acids” [1]. These compounds predominantly originate from the phenylpropanoid acetate pathway and the phenylpropanoid pathway [2]. The phenylpropanoid acetate pathway mainly produces flavonoids with a C6-C3-C6 core structure, whereas representative compounds produced by the phenylpropanoid pathway are lignans typical of a C6-C3 structure [3]. Flavonoids have a variety of roles, such as visual attractants, feeding repellents, photoreceptors, egg-laying stimulants, chemosensitizers, plant antitoxins, antioxidants, and antimicrobials [4]. Meanwhile, lignans are heterogeneous aromatic polymers that integrate with cellulose and hemicellulose to form the secondary cell wall, fulfilling critical physiological functions in water conductance and mechanical support [5]. Upregulation of multiple enzymes associated with the phenylpropanoid acetate or phenylpropanoid pathway has been demonstrated to enhance plant tolerance to biotic stresses, like *Pseudomonas syringae*, *Erwinia carotovora*, *Botrytis cinerea*, and so on [6,7]. This defense reprogramming is often embedded within broader phytohormone signaling networks. In particular, the ethylene and jasmonate pathways are widely recognized as central regulators of defense against necrotrophic pathogens like *B. cinerea*, and these pathways frequently interact in an antagonistic manner with salicylic acid signaling, which is pivotal against biotrophic pathogens [8]. Meanwhile, the activation of these hormone pathways integrates with reactive oxygen species (ROS) signaling, where hormone cues can regulate the oxidative burst, and ROS in turn can influence hormone pathways, forming a complex regulatory network that coordinates plant immunity [9].

Cinnamyl alcohol dehydrogenase (CAD) is the enzyme that catalyzes the key and final step in lignin monomer synthesis, alongside NADPH as a cofactor to reduce pine aldehyde, mustard aldehyde, and ρ-coumarin aldehyde into their corresponding alcohols [10]. The *CAD* gene is found not only in higher plants but also in several symbiotic fungi and some algal species. The expression of *CAD* affects multiple biological processes, such as lignin content, vessel density, seed germination, and resistance to biotic stresses [10]. For instance, the transgenic line of *DkCAD*-overexpression has been demonstrated to enhance anthracnose resistance in persimmon [11]. In addition, *TaCAD12* confers protection against the invasion of *Phytophthora graminis* by regulating the expression of defense genes as well as related genes in the phenylpropanoid pathway in wheat [12]. Moreover, *EgCAD1* promotes lignin biosynthesis under pathogen invasion in *Elaeis guineensis* [13].

The expression of *CAD* in vascular plants is modulated by a diverse array of regulatory factors, encompassing both external stimuli and intrinsic signaling pathways. For instance, *CAD* expression could be activated by gibberellin treatment [14]. In addition, transcription of *CAD* could be regulated by NACs, WRKYs, or other transcription factors to enhance plant response to various signals, such as *Rhizoctonia solani Kühn*, sheath blight disease, and *Colletotrichum horii* [15,16]. Furthermore, PtrCAD1 interacted with cinnamoyl-CoA reductase (CCR), leading to a reciprocal reduction in CAD activities in *Populus trichocarpa* [17]. MeCAD15 physically interacted with MePOD12 in regulating postharvest physiological deterioration via increasing antioxidant capacity and lignin content in Cassava [18]. Taken together, the regulatory mechanisms of altered CAD enzyme activity could be influenced at the pre- and post-transcriptional levels.

As a non-vascular plant, from aquatic green algae to terrestrial plants, moss occupies a special position in plant evolution. *Physcomitrium patens* has emerged as an exceptional model system for investigating evolutionary developmental biology, owing to its high-frequency homologous recombination, well-annotated genome, short life cycle, and other advantageous features [19]. Although *P. patens* possesses an expanded suite of monolignol biosynthesis genes compared to green algae and represents the first organism with a full genetic complement for this pathway, there is a controversy about the presence of lignan monomers in *P. patens* [20]. By relatively analyses of secondary metabolites in gametophores of 4-coumaroyl-shikimate 3′-hydroxylase (*PpCYP98*) knockout and wild-type lines in a previous study, it is putative that *P. patens* harbors the potential to synthesize pre-lignin compounds [21,22,23]. In addition, four putative *CADs* were identified through comprehensive genome annotation of *P. patens*. Previous studies have shown that *PpCAD1* is most highly expressed in gametophores and influences moss architecture. The phenolic compounds were enhanced in the cell walls of *PpCAD1*-overexpressing gametophore, conferring resistance to *Botrytis cinerea*. Meanwhile, heterologous expression in *Arabidopsis* elevated G-lignin, promoting seedling resistance through structural barriers against fungal pathogenesis [24]. Contrastingly, *PpCAD4* showed minimal gametophytic expression but exhibited pronounced transcriptional activation post-*B. cinerea* challenge compared to *PpCAD1*. However, the detailed functions and the regulatory mechanism of *PpCAD4* were still obscure.

In this study, we found that *PpCAD4* enhanced the resistance of *P. patens* after *B. cinerea* infection. In addition, integrative transcriptomic and metabolomic results further determined that *PpCAD4* was able to increase the expression of genes and metabolites correlated with the phenylpropanoid acetate and the phenylpropanoid pathway after *B. cinerea* treatment. Finally, we identified candidate transcriptional regulators and protein interactors, uncovering the potential regulatory mechanism of *PpCAD4* in early terrestrial plants against biotic stresses. These findings contribute to uncovering the molecular mechanism of defense underlying the enzyme of lignin biosynthesis pathway in non-vascular terrestrial moss, and provide a potential target for enhancing the resistance of crops to necrotic pathogens.

## 2. Results

### 2.1. PpCAD4 Promoted Resistance of Gametophore Against B. cinerea Assault

**Figure 1:**
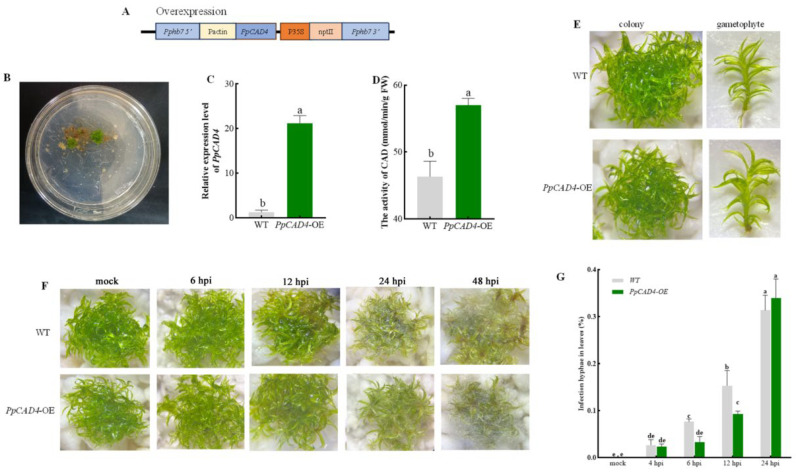
Upregulation of *PpCAD4* promoted resistance of gametophore against *B. cinerea* assault. (**A**) Schematic outline of the *PpCAD4*-OE vector. The primers used for identifying overexpression lines are marked. Pactin: promoter of rice actin; 35S: cauliflower mosaic virus 35S promoter. (**B**) Screening of *PpCAD4*-OE transgenic lines using 50 μg/mL of geneticin G418, bar = 2 cm. (**C**) Relative expression levels of *PpCAD4*. (**D**) Enzyme activity assays of CAD. (**E**) Morphological comparison of colonies and gametophores, bar = 2 mm. (**F**) Disease symptoms observed in *B. cinerea*-infected gametophores of wild-type and *PpCAD4*-OE lines at various hours post inoculation (hpi), bar = 1 cm. (**G**) Infection hyphae in leaves. Data were presented as mean ± SD; different letters indicate significant differences at *p* < 0.05.

An overexpression vector containing the coding sequence of *PpCAD4* under the stronger induction of the rice actin promoter was constructed, resulting in the disruption of *Pphb7* (Figure 1A). We transferred the recombinant vector pTFH15.3-*PpCAD4* mediated by PEG into the protoplast. After two weeks of selection for G418 resistance encoding by *nptII* induced by CaMV35S, transformants were then grown on normal PpNH_4_ medium for further cultivation (Figure 1B). To confirm the presence of the overexpression cassette, three primers, including *PpHB7*, *Pp15.3up*, and *Pp15.3down*, were used to amplify the various regions in *PpCAD4*-OE and wild type, confirming the successful generation of *PpCAD4*-OE positive gametophores (Figure S1). In addition, qRT-PCR quantification of gametophores revealed a 23-fold increase in *PpCAD4* transcript levels, and further assessment of CAD enzyme showed that its activity was increased by 21.74% consistently in transgenic lines (Figure 1C,D). In terms of moss colony and gametophore, no significant changes were observed between *PpCAD4*-OE and wild type, different from the morphology of *PpCAD1*-OE lines [24] (Figure 1E). Furthermore, qRT-PCR showed that the other three *PpCADs* homologs, *PpCAD1*, *PpCAD2*, and *PpCAD3*, displayed basically no alteration in *PpCAD4*-OE lines compared to wild type (Figure S2).

To gain more insights into the influence of *PpCAD4* after pathogen attack, the gametophores of *PpCAD4*-OE and WT were inoculated with *B. cinerea* at different times (Figure 1F). At 12 hpi, significant brown maceration regions appeared at the tips of the phyllid in the WT, whereas only discernible symptoms of brown spots were observed in *PpCAD4*-OE at the same period. At 24 hpi, hyphae were observed and almost surrounded by the whole gametophores of wild type, whereas fewer hyphae and a verdant phenotype were noticed in the colony of overexpression lines. With the aggressive fungal invasion at 48 hpi, hyphae proliferation and tissue colonization appeared in both genotypes. To precisely reflect the infection condition, the number of hyphae-infected cells was counted and displayed that the infection hyphae increased gradually with inoculation time (Figure 1G). No remarkable differences occurred between the two genotypes at 4 hpi. However, the significantly divergent values of infected hyphae were visible at 6- and 12 hpi between *PpCAD4*-OE lines and the wild type. Finally, no significant divergences were again observed at 24 hpi, due to the majority of moss cells being invaded and filled with hyphae of *B. cinerea*, suggesting that the defense responses occurred at the early stages.

### 2.2. The Flavonoid, Phenylpropanoid Pathway and Plant Hormone Signal Transduction Were Enhanced in PpCAD4-OE After B. cinerea Assault

**Figure 2:**
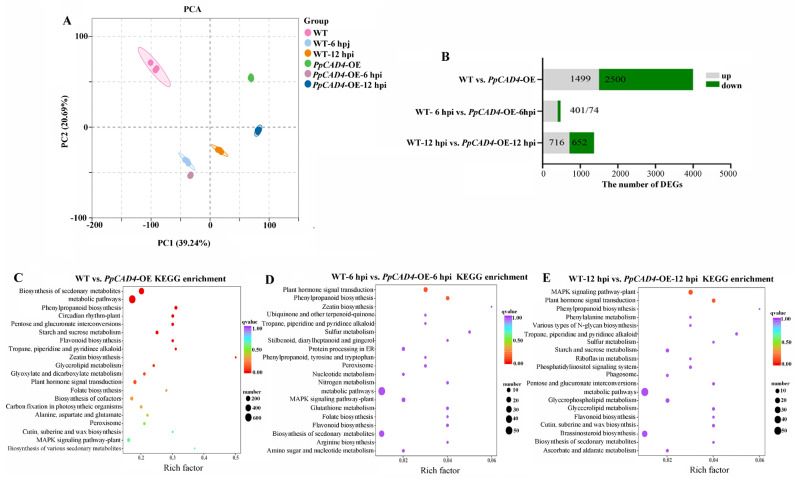
Overexpression of *PpCAD4* influenced the gene associated with the flavonoid, phenylpropanoid pathway, and plant hormone signal transduction. (**A**) Principal component analysis (PCA) of DEGs. (**B**) Number of DEGs. (**C**–**E**) KEGG enrichment of the DEGs between WT and *PpCAD4*-OE (**C**), at 6 hpi (**D**), and 12 hpi (**E**).

To elucidate the detailed functions of *PpCAD4* in response to biotic stress, RNA-sequencing was performed on gametophores of WT and *PpCAD4*-OE following treatment with *B. cinerea* at 0-, 6-, and 12 hpi, respectively. The base quality scores (Q30) for all eighteen samples surpassed 93%. In addition, approximately 67.89% to 97.65% of clean reads were successfully mapped to the *P. patens* reference genome (Table S1). Principal component analysis (PCA) revealed that the two components, PC1 and PC2, respectively, accounted for 55.24% and 20.69% of the total variance (Figure 2A). Comparative transcriptomic analysis identified 3999, 475, and 1368 differentially expressed genes (DEGs) in *PpCAD4*-OE compared to wild type at 0-, 6-, and 12 hpi, respectively. Among these, 1499, 401, and 716 DEGs were individually upregulated, whereas 2500, 74, and 652 DEGs were downregulated at the corresponding time points (Figure 2B, Table S2).

Functional analysis indicated that *PpCAD4* modulated the primary metabolism (starch and sucrose metabolism, pentose and glucuronate interconversions, glycerolipid metabolism and glyoxylate and dicarboxylate metabolism), secondary metabolism (phenylpropanoid and tropane, piperidine and pyridine alkaloid biosynthesis, flavonoid biosynthesis), as well as influencing plant signals (plant hormone signal transduction, zeatin biosynthesis) in *PpCAD4*-OE lines (Figure 2C, Table S3). At 6 hpi, DEGs in *PpCAD4*-OE lines were predominantly enriched in phenylpropanoid biosynthesis and plant hormone signal transduction (Figure 2D, Table S3). By 12 hpi, significant enrichment was observed in the mitogen-activated protein kinase (MAPK) signaling pathway, plant hormone signal transduction, and phenylpropanoid biosynthesis became apparent (Figure 2E, Table S3). Overall, transcriptomic profiling demonstrated that *PpCAD4* pronouncedly influenced gene expression with flavonoid, phenylpropanoid biosynthesis, and plant hormone signaling pathways. In addition, the results showed a similar trend between RNA-Seq and qRT-PCR data, confirming the reliability of RNA-Seq data (Figure S2).

### 2.3. PpCAD4 Promoted the Accumulation of Flavonoids and Phenolic Acids After B. cinerea Attack

**Figure 3:**
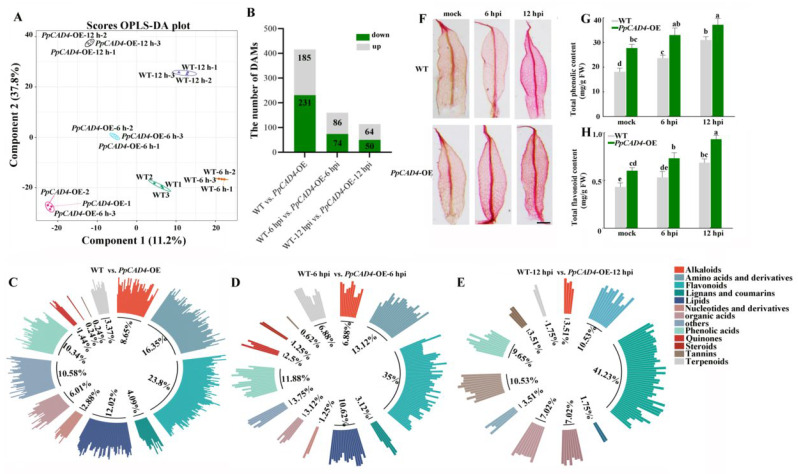
Enhanced *PpCAD4* expression triggered the accumulation of phenolic and flavonoid compounds. (**A**) The OPLS-DA score plot comparing WT and *PpCAD4*-OE lines under different *B. cinerea* infection time. (**B**) Numbers of differently accumulated metabolites (DAMs). (**C**–**E**) The class of DAMs composition distribution in WT VS. *PpCAD4*-OE, at 6 hpi (**D**), and 12 hpi (**E**). (**F**) Cytological observation of phyllids stained using safranin-O. Bar = 1 mm. (**G**) Total phenolic and (**H**) flavonoid content in wild type and *PpCAD4*-OE lines after *B. cinerea* inoculation. Data were presented as mean ± SD; different letters indicate significant differences at *p* < 0.05.

UPLC-MS/MS was utilized for metabolomic profiling of WT and *PpCAD4*-OE samples at 0-, 6-, and 12 hpi following *B. cinerea* infection. The orthogonal partial least squares discriminant analysis (OPLS-DA) model effectively differentiated WT and *PpCAD4*-OE (Figure 3A). There were 2065 differential metabolites determined, comprising 418 flavonoids, 306 phenolic acids, 201 lipids, 296 amino acids, and their derivatives (Figure S4A).

A total of 416 metabolites exhibited significant alterations in *PpCAD4*-OE compared with WT, and *PpCAD4*-OE exhibited changes in the number of significantly altered metabolites, reaching 160 and 114 at 6- and 12 hpi, respectively (Figure 3B, Table S4). We observed that the total proportion of differential accumulation metabolites (DAMs) associated with phenolic acid and flavonoids in *PpCAD4*-OE was 34.14%, 46.88%, and 50.88% in at 0-, 6-, and 12 hpi, respectively (Figure 3C–E). KEGG pathway analysis revealed a significant enrichment of pathways associated with flavonoid biosynthesis (Figure S4B–D, Table S5). Moreover, the results of safranin-O staining and total phenolic content indicated that *PpCAD4* could increase the content of phenolic acids under *B. cinerea* infection, speculating that phenolic acids might be a significant defense response (Figure 3F,G). *PpCAD4*-OE lines exhibited a marked increase in total flavonoid content compared to WT (Figure 3H). Moreover, the activities of PAL and POD enzymes involved in the phenylpropanoid pathway showed a significant increase. After *B. cinerea* infection, the qPCR assay manifested that the promotion of six genes implicated in phenylpropanoid pathways in *PpCAD4*-OE at 6 and 12 hpi (Figure S5).

### 2.4. The Integrated Analysis of Transcriptome and Metabolism Between PpCAD4-OE and WT

**Figure 4:**
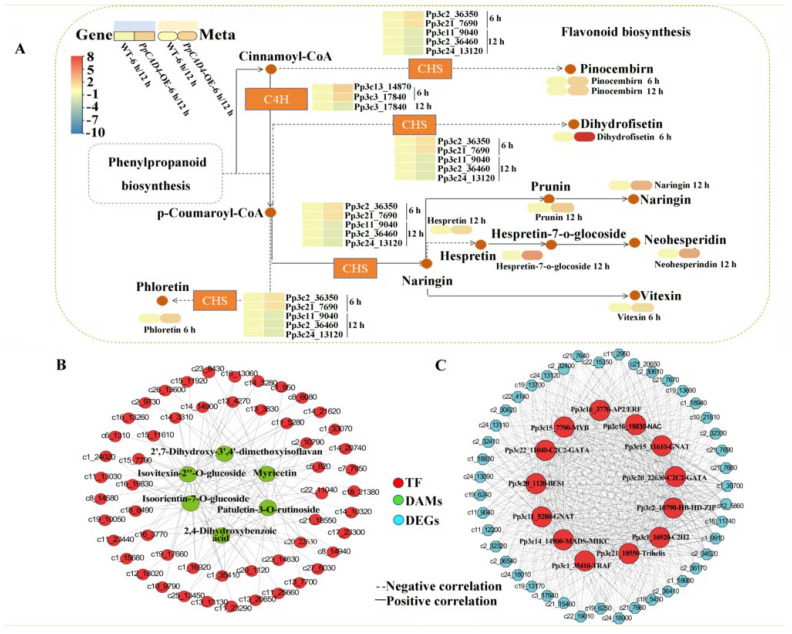
Integrated analysis of metabolomic and transcriptomic data involved in flavonoid and phenylpropanoid biosynthesis pathways in *P. patens* under *B. cinerea* treatment. (**A**) Transcript and metabolite abundances at 6- and 12 hpi. (**B**) Interaction network between regulatory transcription factors (red circles) and the top ten enriched phenolic acids and flavonoids (green circles). (**C**) Coordinated regulatory network of TFs (red circles) and genes (blue circles) governing phenylpropanoid and flavonoid biosynthesis pathways.

To investigate the association between induced DEGs and DAMs under *B. cinerea* stress, the KEGG analysis indicated that the pathway related to flavonoid biosynthesis (Figure S6 and Table S6), suggesting that the flux through these metabolic pathways increased in response to *B. cinerea*. In addition, alpha-linolenic and linoleic acid metabolism were also enriched. At 6 hpi, the flavonoid compounds, including phloretin, vitexin, dihydrofisetin, and pinocembrin were increased by overexpression of *PpCAD4* (Figure 4A). In addition, the concentrations of pinocembrin, prunin, naringin, hesperetin, hesperetin-7-O-glucoside, hespretin-7-O-glocoside and neohesperidin were accumulated in *PpCAD4*-OE-12 hpi despite the decreased expression level of *CHS*. Consequently, this relationship suggests that alterations of DEG in *PpCAD4*-OE promote flavonoid accumulation, which enhances plant resilience to *B. cinerea* stress.

To determine the key regulatory network implicated in “phenolic acids” influenced by *PpCAD4*, major transcription factors were then identified using differentially expressed TFs and metabolites between WT and *PpCAD4*. A correlation analysis was conducted to establish relationships between 116 TFs and the top 10 increased metabolites in phenolic acids and flavonoids, with a positive correlation coefficient greater than 0.8 within WT and *PpCAD4*-OE. Among these genes and metabolites, 116 TFs showed strong correlations with 6 phenolic acids and flavonoid-related metabolites (Figure 4B). Consequently, a regulatory network was constructed between these TFs and structural regulatory genes involved in phenolic acids and flavonoids biosynthesis (Figure 4C). The top five TFs associated with the biosynthesis of phenolic acids and flavonoids were individually GNAT (*Pp3c11_5280*), MADS-MIKC (*Pp3c14_14900*), NAC (*Pp3c16_19830*), C2H2 (*Pp3c1_16920*), and C2C2-GATA (*Pp3c20_22630*) (Table S7). Furthermore, among the phenolic acids and flavonoids-related metabolites, isovitexin-2″-O-glucoside, isoorientin-7-O-glucoside, patuletin-3-O-rutinoside, myricetin, and 2, 4-Dihydroxybenzoic acid emerged as the most significantly different phenolic acids and flavonoids in *P. patens* (Table S7). These results indicated that *PpCAD4* might be regulated by GNAT, MADS-MIKC, NAC, and C2H2 in defense response.

### 2.5. PpCAD4 Could Be Negatively Regulated by PpMC6 In Vitro and Vivo

**Figure 5:**
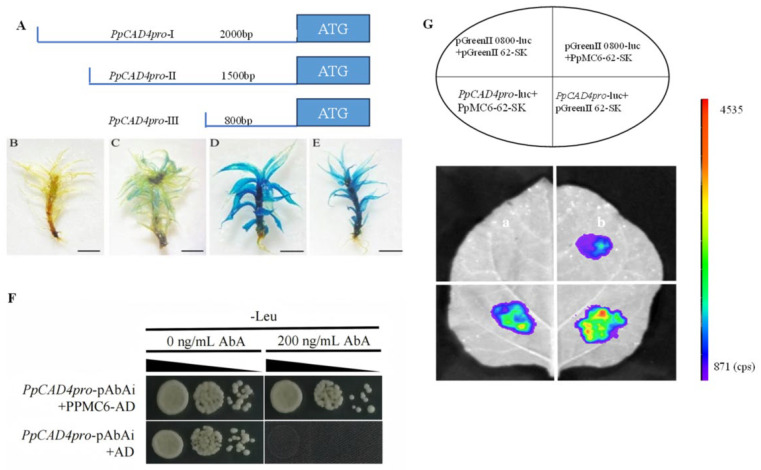
PpMC6 directly regulated the transcription of *PpCAD4*. (**A**) Schematic representation of the ~2000 bp promoter region of *PpCAD4*. (**B**–**E**) GUS activity in transgenic lines harboring *PpCAD4pro*::GUS constructs with promoter fragments of ~800 bp (**C**), ~1500 bp (**D**), and ~2000 bp (**E**), compared to the wild type (**B**), bar = 1 cm. (**F**) Yeast one-hybrid (Y1H) assay demonstrating the interaction between PpMC6 and the *PpCAD4pro*. (**G**) Luciferase (LUC) reporter assay showing promoter activity driven by the *PpCAD4pro*; red and purple signals indicated strong effector-reporter binding affinity.

To determine the critical regulatory regions of the *PpCAD4* promoter, we performed GUS histochemical assays by generating pTFH15.3-*PpCAD4pro*::GUS transgenic lines using homologous recombination. Promoter fragments of varying lengths, designated *PpCADpro*-I, -II, and -III, are illustrated in Figure 5A. Transformation with the empty vector yielded no detectable GUS signal (Figure 5B), whereas the approximately 800 bp promoter fragment exhibited minimal GUS activity (Figure 5C). In contrast, the ~1500 bp (Figure 5D) and ~2000 bp (Figure 5E) promoter fragments drove broad GUS expression throughout *P. patens*, demonstrating comparable transcriptional activity.

To identify the transcription factors that bound to *PpCAD4pro*, we leveraged the transcriptionally active regions of *PpCAD4* as bait in a Y1H screening. The bait-reporter yeast strain was transformed with a *P. patens* cDNA library, and 111 prey clones were obtained (Table S8). After sequencing the prey plasmids, we found that 6 clones were designated as transcription factors, and 5 of them were annotated as the same transcription factor. After further investigation, PpMC6 (*Pp3c13_24590*), belonging to the MADS-box transcription factor, was determined, consistent with the above-constructed regulatory network. Specifically, the CDS of *PpMC6* was cloned into the pGADT7 vector for Y1H assays, utilizing combinations of bait and prey vectors. These results suggested that PpMC6 could control the expression of *PpCAD4* via binding to the promoter directly (Figure 5F). To validate the direct regulation of PpMC6 in its target transcripts, a dual-luciferase transient expression assay was conducted in tobacco leaves. The results indicated that the presence of the PpMC6 significantly suppressed LUC activity, corresponding to the transcriptional repression of *PpCAD4* (Figure 5G).

### 2.6. PpCAD4 Had Interactions with PpE3 and PpCAT2 Individually

**Figure 6:**
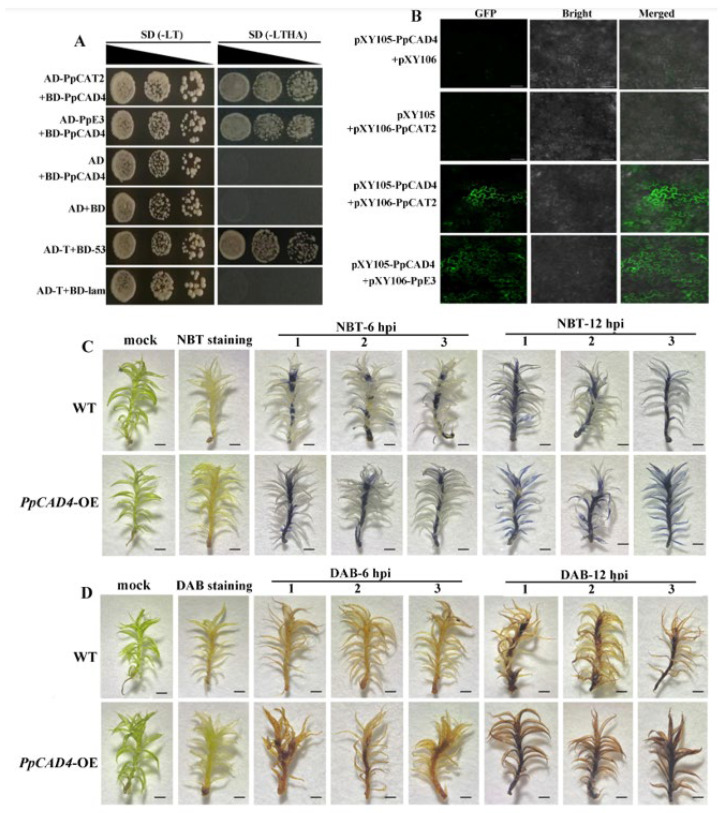
Interaction of PpCAD4 with PpE3 and PpCAT2. (**A**) Physical interactions between PpCAD4 and PpE3 or PpCAT2 in the AH109 yeast strain. SD (-LW), SD medium lacking leucine and tryptophan; SD (-LWHA), SD medium without leucine, tryptophan, histidine, and adenine. (**B**) BiFC assay showed that PpCAD4 interacts with PpE3 and PpCAT2, respectively. Bars = 100 μm. (**C**) Nitroblue tetrazolium (NBT) and (**D**) diaminobenzidine (DAB) staining revealing O_2_^−^ and H_2_O_2_ accumulation in WT and *PpCAD4*-OE, respectively, bar = 1 mm.

To identify potential PpCAD4-interacting proteins, we used the yeast two-hybrid (Y2H) system to screen for interacting proteins from a cDNA library (Table S9). We detected the interaction of candidate proteins PpE3 (*Pp3c8_23280*) and PpCAT2 (*Pp3c22_11690*) by both the Y2H and BiFC. The results showed that the yeast AH109 strain transformed plasmids AD-PpCAD4 with BD-PpE3 or BD-PpCAT2 grew well in the medium (SD-LWHA), indicating that PpCAD4 can interact with PpE3 and PpCAT2 in yeast cells (Figure 6A). Moreover, the BiFC assay confirmed the interaction in *Nicotiana benthamiana*, and the fluorescence signals revealed interactions between PpCAD4 and PpE3 or PpCAT2 (Figure 6B).

**Figure 7:**
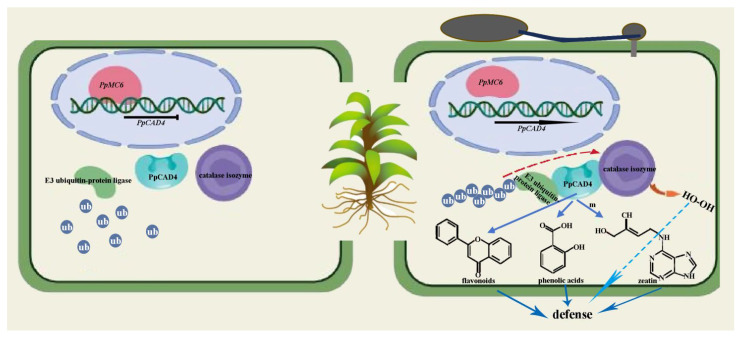
A proposed mechanistic model of *PpCAD4*-mediated immune responses in *P. patens*. *PpCAD4*, significantly upregulated in response to *B. cinerea* infection, is negatively regulated by PpMC6. Enhanced *PpCAD4* expression stimulates the biosynthesis of flavonoids, phenolic acids, and zeatin. PpE3 and PpCAT2 interact directly with PpCAD4, suggesting that PpCAD4 induces PpE3-mediated degradation of catalase isozyme 2 (PpCAT2), leading to increased reactive oxygen species (ROS) production as a defense mechanism against *B. cinerea*.

Additionally, the NBT and DAB results revealed that the gametophore of the *PpCAD4*-OE strains was significantly more stained than that of the WT, suggesting the significant increase in the accumulation of O_2_^−^ and H_2_O_2_ in the gametophore of the *PpCAD4*-OE plants compared to the WT (Figure 6C,D). We speculated that PpCAT2 and PpE3 interact indirectly via PpCAD4, suggesting that PpE3-mediated degradation of PpCAT2 elevated H_2_O_2_ levels to resist necrotrophic pathogen infection. Thus, the defense response of *PpCAD4* in moss against pathogens may be activated not only through alterations in secondary metabolites, but also via the oxidative system involving CAT, akin to the similar defense mechanisms in vascular plants (Figure 7).

## 3. Discussion

Although the presence of lignin in moss remains highly debated, the *P. patens* genome contains orthologs of all essential enzymes involved in lignin biosynthesis. Comparative genomic analyses confirm CAD gene family expansion across plant lineages, with paralogs exhibiting functional diversification in phenylpropanoid metabolism and environmental adaptation [25]. Our findings similarly indicated that phenylpropanoid and flavonoid biosynthesis were identified as two key pathways in *B. cinerea* stress by integrated transcriptome and metabolome analysis in *PpCAD4*-OE and wild type. Interestingly, our study determined that flavonoids displayed a more considerable difference induced by *PpCAD4* than phenylpropanoids after the biotic stress. This evolutionary hierarchy aligns with flavonoid emergence as primordial phenolic defenses in land plants, predating the acquisition of monolignol and lignin pathways [26]. This means that flavonoids might play a more significant role in defense responses in early terrestrial plants to conquer land.

Gene expression profiling revealed divergent transcriptional dynamics between *PpCAD1* and *PpCAD4* during gametophore morphogenesis and pathogen defense signaling in *P. patens*, suggesting that these paralogs likely mediate distinct biological functions. The divergence of CAD likely originates from evolutionary developmental demands, with specific genes essential for monolignol biosynthesis-regulated plant growth, while others govern environmental adaptation [24]. Our previous study showed that *PpCAD1*-OE lines displayed an elongated stem and a loosely organized architecture, whereas *PpCAD4*-OE lines exhibited no discernable alterations. In addition, *PpCAD4* exhibited significant up-regulation in *P. patens* under *B. cinerea* infection, whereas *PpCAD1* showed negligible transcriptional activity, suggesting that *PpCAD4* might play a major role in plant defense, rather than moss architecture [24]. Thus, the functional divergence of the CAD family exemplifies the adaptive mechanisms to confront the challenges of terrestrial adaptation in moss.

Our study found that PpMC6, a member of the MADS family, might regulate *PpCAD4* expression by Y1H and LUC assays. The MADS gene family, ubiquitously present in eukaryotes, features a conserved MADS domain at the N-terminus, encoded by 56–60 amino acids, and plays an important role in plant growth and development, as well as defense responses. In *Prunus persica*, *PpMADS2* potentiated the transcription of a group of pathogenesis-related genes and conferred fungal resistance, such as against *Rhizopus stolonifer*, leading to a significant delay in the symptomatic appearance of disease [27]. Likewise, MADS51 activates MIR1863a in leaves and panicles of rice upon pathogen invasion [28]. PpMC6 has been proven to facilitate external water conduction during drought stress in *P. patens*, while the development of water-conducting cells was associated with the phenylpropanoids during terrestrial adaptation [29,30]. Our work also proved that PpMC6 negatively regulated *PpCAD4,* which encodes a key enzyme in the phenylpropanoid pathway crucial for lignin-like compound biosynthesis, speculating that PpMC6 might enhance the ability of non-vascular plants to adapt to terrestrial environments, including water stress resilience and pathogen attack.

Here, we identified that PpCAD4 interacted with PpE3 and PpCAT2, respectively. Within the ubiquitination system, E3 ubiquitin ligase proteins play essential roles in the recognition of substrates in protein degradation, regulating various biological processes, especially in plant defense response. For example, degradation of COP9 signalosome by the E3 ligase enhanced resistance against the fungal pathogen *Magnaporthe oryzae* and the bacterial pathogen *Xanthomonas oryzae* without affecting rice growth [31]. CAT, a core antioxidant enzyme in most organisms, is critical in physiological processes, including plant growth and development, pathogen defense, and oxidative aging [32]. In *Arabidopsis*, the knockout mutant of *AtCAT2* exhibits enhanced resistance to biotrophic pathogens and heightened susceptibility to necrotrophic pathogens [33]. Previous studies demonstrated that the rice E3 ubiquitin ligase APIP6 interacted with catalase OsCAT, facilitating H_2_O_2_ accumulation through targeted protein degradation, thereby amplifying ROS-triggered defense signaling cascades [34]. Our study demonstrated that PpCAT2 and PpE3 can interact with PpCAD4, placing them within the same protein interaction network. Critically, our histochemical data revealed a significant increase in O_2_^−^ and H_2_O_2_ accumulation specifically in the *PpCAD4*-OE lines upon infection. Given that CAT enzymes are key regulators of H_2_O_2_ homeostasis, the observed ROS burst, combined with the interaction network, leads us to propose a testable model: PpCAD4 may scaffold an interaction between PpE3 and PpCAT2, potentially leading to PpE3-mediated modulation of PpCAT2 activity or stability. This could, in turn, result in the elevated H_2_O_2_ levels that contribute to the enhanced defense phenotype against necrotrophic pathogens. Therefore, the defense mechanism of *PpCAD4* in *P. patens* may involve a dual strategy: not only reprogramming secondary metabolism but also modulating the oxidative system via CAT, reminiscent of defense integrations in vascular plants.

## 4. Materials and Methods

### 4.1. Plant Material and Inoculation Observation

The ecotype Gransden 2004 of *P. patens* served as the WT strain, which is the genetic background for all transgenic lines. Gametophores were routinely cultivated on PpNH4 medium at 25 °C under a photoperiod cycle of 16 h of light alternating with 8 h of darkness [21]. *B. cinerea* was cultivated in Petri dishes in potato dextrose agar (PDA) medium (25 °C). The spore suspension underwent sequential filtration through sterile nylon gauze (200 μm mesh) to eliminate contaminants, while potato dextrose broth (PDB) was utilized as a mock. Following adjustment of conidial density to 1 × 10^5^ spore/mL for subsequent analysis, the spores were deposited onto the gametophores of *P. patens* [35]. The rate of hyphal infection was quantified at 4-, 6-, 12-, and 24- h post inoculation (hpi) using light microscopy (BX-61, Olympus, Tokyo, Japan).

### 4.2. Vector Construction and PpCAD4-OE Lines Generation

The full-length coding sequence (CDS) of *PpCAD4* (1278 bp) was PCR amplified with specific primers (Table S10). To create the vector of *PpCAD4*-OE for moss transformation, the CDS of *PpCAD4* was inserted into vector pTFH15.3 (provided by Capital Normal University, Beijing, China) and linearized using *Not* I. Subsequently, this linearized plasmid was applied for polyethylene glycol (PEG)-mediated transgenic transformation. After 5–7 days of cultivation, protoplasts of *P. patens* were isolated using 1% driselase (Sigma, St. Louis, MO, USA) from protonema, and the density of protoplast suspension was quantified using a hemocytometer. PEG-mediated transformation was carried out by incubating 1.6  ×  10^6^ protoplasts/mL with approximately 30 μg linearized DNA. Stable transgenic lines were selected through two successive rounds of cultivation on media containing the antibiotics G418 and further PCR validation.

### 4.3. Quantitative Real-Time PCR (qRT-PCR) Assays

RNA extraction and quantitative PCR were performed as described previously [36]. Total RNA from *P. patens* protonema was isolated using RNA Extraction Kit (Promega, Shanghai, China), and cDNA was then synthesized using Tiangen reverse transcription KR118-02 kit (Tiangen, Beijing, China). The qRT-PCR was conducted using the Roche LightCycler480 system with SYBR qPCR Master Mix (Vazyme Biotech, Nanjing, China). The relative primers were listed in Table S10. The cycling parameters include an initial denaturing of cDNA template at 95 °C for 30 s, followed by 40 cycles of denaturing at 95 °C for 10 s and annealing/extension at 60 °C for 30 s. The mix proportions and cycling parameters were implemented in accordance with the manufacturer’s protocol. The expression values relative to the control were evaluated using the 2^−△△Ct^ method. Three biological replicates were analyzed to ensure experimental reproducibility and reliability.

### 4.4. Measurement of Enzyme Activity

The enzymatic activities of CAD, phenylalanine ammonia-lyase (PAL), and peroxidase (POD) were assayed using the BC4170, BC0210, and BC0090 kits (Solarbio, Beijing, China). Assays were performed using 0.1 g of *P. patens*. Following the addition of an adequate volume of pre-cooled extraction solution, the gametophore was homogenized into a fine slurry on ice. This slurry was centrifuged at 10,000× *g* for 20 min at 4 °C. Absorbance readings were obtained at the respective wavelengths after termination of the solution. Enzyme activities, calculated by comparing sample absorbance values against the standard curve, are expressed in mmol/g. All samples were analyzed with a minimum of three biological replicates to ensure the reliability and reproducibility of the data.

### 4.5. RNA Sequencing and Transcriptome Analysis

Gametophores from WT and *PpCAD4*-OE lines were inoculated with *B. cinerea* at 0-, 6-, and 12 hpi and collected. Each infected treatment was assessed with three independent biological replicates. The cDNA library was prepared using an Illumina HiSeq 3000 platform to produce 150 bp paired-end reads (Metware, Wuhan, China). The trimmed reads based on Hisat2 were mapped to the *P. patens* genome (https://www.ncbi.nlm.nih.gov/data-hub/genome/GCF_000002425.4/, accessed on 29 December 2025) [37]. Data normalization and differential expression analysis were carried out following the DESeq2 [38]. Differentially expressed genes (DEGs) were identified under the adjusted *p*-value of false discovery rate ≤ 0.01 and abs [log2(Fold change)] ≥ 1 using KOBAS 3.0 software [39]. The pathway enrichment analysis was performed using Kyoto encyclopedia of genes and genomes (KEGG database, http://www.genome.jp/kegg/, accessed on 29 December 2025).

### 4.6. Widely Targeted Metabolome Experiment and Correlation Analysis of Transcriptome and Metabolome

The biological samples of the phyllids were placed in a lyophilizer and pulverized into a fine powder. A 50 mg aliquot of the powder was extracted using 70% methanol at 4 °C. After centrifugation, the resulting extract was subsequently filtered for ultra-performance liquid chromatography-tandem mass spectrometry (UPLC-MS/MS) analysis [40]. UPLC-MS/MS data acquisition and processing were performed following established analytical protocols [41]. Metabolite identification and quantification were performed utilizing the Metware Database (Wuhan Metware Biotechnology, Wuhan, China), and the detailed methods are described in Supplementary Note S1. Utilizing the KEGG database, the differential metabolites between *PpCAD4*-OE and WT lines were analyzed. Co-expression correlation coefficients were computed using R 4.3.0 software [42]. Pearson correlation coefficient abs (PCC) ≥ 0.8 and the *p*-value ≤ 0.5 were chosen. Functional relationships between transcription factors (TFs) and phenolic acids, flavonoids, or genes involved in flavonoid and phenylpropanoid biosynthesis were mapped using Cytoscape 3.8.0 (https://cytoscape.org/, accessed on 29 December 2025).

### 4.7. Measurement of Total Flavonoids and Phenols

Total flavonoids and total phenols were determined by commercially available kits (G0118W, G0117W; Suzhou Grace Biological Company, Suzhou, China). All samples were analyzed with a minimum of three biological replicates to ensure the reliability and reproducibility of the data. The phyllids of WT and *PpCAD4*-OE were stained with safranin-O to evaluate the accumulation of phenolic compounds [21]. Imaging was observed under an Olympus BX61 microscope (Olympus, Tokyo, Japan).

### 4.8. GUS Activity Assay

The 2000 bp promoter region upstream of the *PpCAD4* initiation codon was PCR amplified from genomic DNA and subsequently cloned into the pTFH15.3::GUS to drive the expression of the GUS reporter gene. The linearized plasmid of *PpCAD4_Pro_*::GUS was transmitted into the protoplasts mediated by PEG, and empty pTFH15.3::GUS was used as a positive control. Histochemical analysis of β-Galactosidase (GUS) activity was performed utilizing a standardized GUS Stain Kit (Solarbio, Beijing, China). All primers utilized for the GUS activity assay are detailed in Table S10.

### 4.9. Construction of P. patens cDNA Library in Yeast

The construction of the *P. patens* cDNA library in yeast was used RNA isolated from gametophores. The primary library yielded a clone count of 8.17 × 10^6^ cfu. Plasmid DNA from the primary library was electroporated into *DH10B* cells to produce the secondary library, which achieved a clone count of 1.27 × 10^7^ cfu. The methodology aligns with previously reported [43].

### 4.10. Yeast One-Hybrid Screening and Assay (Y1H)

We amplified the sequences of *PpCAD4pro* from *P. patens* genomic DNA and cloned them into the bait pAbAi vector (Clontech, San Jose, CA, USA), linearized, and subsequently transformed into Y1HGold cells according to the previous methods described in Supplementary Note S1 [44]. Colonies were isolated from the synthetic dextrose (SD) medium. After identifying the minimal inhibitory concentration of aureobasidin A (AbA), the *P. patens* cDNA library was introduced into yeast cells and selected using SD/-Leu/AbA. Y1H assay was afterwards carried out to determine the interactions of the putative TF with *PpCAD4pro*. Both were transformed and cultured on SD/-Leu/AbA media.

### 4.11. Luciferase Complementation Assay

The *PpMC6* CDS was inserted into the effector vector pGreenII 0800 62-SK plasmid, while the *PpCAD4pro* was inserted into the reporter pGreenII 0800-LUC vector. Both constructs, along with an empty-vector control, were individually introduced into *Agrobacterium tumefaciens* strain GV3101 carrying the pSoup plasmids. Each combination composed of two different vectors was performed by injecting them into *N. benthamiana* leaves as reported previously [45]. All primers were listed in Table S10, and each experiment was performed with a minimum of three biological replicates.

### 4.12. Yeast Two-Hybrid Screening and Assay (Y2H)

A cDNA library from *P. patens* was screened using the pGBKT7 (BD) vector containing the *PpCAD4* coding sequence. The BD-PpCAD4 plasmids and candidate interaction proteins cloned into pGADT7 vectors were co-transfected into AH109 yeast cells. Empty vectors were co-transformed as negative controls. All primers were provided in Table S10.

### 4.13. Bimolecular Fluorescence Complementation (BiFC) Assays

The coding sequences of *PpCAD4* and its putative interacting proteins were individually inserted into the pXY105 (cYFP) and pXY106 (nYFP) vectors for BiFC analysis. These plasmids were, respectively, transformed into *A. tumefaciens* strain GV3101 and various combinations of cYFP and nYFP were co-infiltrated into *N. benthamiana* leaves as previously described [46]. After infiltration for 48 h, the YFP fluorescence signals were captured and visualized using a confocal microscope (Leica, Wetzlar, Germany). Primer sequences were provided in Table S10.

### 4.14. Histochemical Detection of Reactive Oxygen Species (ROS)

The *P. patens* were inoculated with *B. cinerea* and harvested at 6 and 12 hpi for ROS detection. For superoxide anion visualization, phyllids were vacuum-infiltrated with 1 mg/mL nitroblue tetrazolium (NBT) in 10 mM sodium azide and 10 mM phosphate buffer (pH 7.5), followed by incubation at room temperature for 30 min. The NBT solution was then removed, and the tissues were cleared by boiling in 95% ethanol for 10 min to decolorize completely. For hydrogen peroxide detection, the cleared tissues were incubated in 1 mg/mL 3,3′-diaminobenzidine (DAB) solution in the dark at 25 °C for 2 h. Subsequently, the DAB solution was aspirated, and the tissues were again cleared by boiling in 95% ethanol for 10 min until all chlorophyll was removed. Finally, the phyllids were preserved in anhydrous ethanol for microscopic observation.

### 4.15. Statistical Analysis

All experimental results are presented as mean ± standard deviation (SD) (*n* = 3). Data were analyzed and displayed using GraphPad Prism (GraphPad Software Inc., version 10, San Diego, CA, USA). The one-way analysis of variance (ANOVA) followed by Dunnett’s test was used to determine the statistical significance. The significant difference test (*p* < 0.05) was employed and considered statistically significant.

## 5. Conclusions

Our study identified *PpCAD4* as the major contributor to defense responses against *B. cinerea* within the *PpCAD* gene family. Furthermore, *PpCAD4* was demonstrated to enhance the phenylpropanoid-acetate and phenylpropanoid biosynthetic pathways through transcriptome and metabolome, thereby orchestrating defense mechanisms against *B. cinerea* infection. We additionally revealed a transcription factor, PpMC6, which negatively regulated *PpCAD4*. Finally, the individual interactions of PpE3 and PpCAT2 with PpCAD4 suggest a possible mechanism for their involvement in the *PpCAD4*-mediated defense against *B. cinerea* in *P. patens*. Therefore, these findings offer new insights into the defense mechanisms of *PpCAD4* in ancestral plants against pathogen invasion.

## Data Availability

The datasets generated in this study have been deposited in the NCBI (https://www.ncbi.nlm.nih.gov/) database, specifically under *PpCAD4* (GenBank number: XM_024531924.1). RNA-Seq raw data from eighteen samples were deposited in the National Center for Biotechnology Information (NCBI) under the accession number (Bioproject ID: PRJNA1210674 and SRA ID: SRR32057684-SRR32057701).

## Supplementary Materials

The following supporting information can be downloaded at: https://www.mdpi.com/article/10.3390/plants15030413/s1.

## Abbreviations

The following abbreviations are used in this manuscript:

CAD	Cinnamyl alcohol dehydrogenase
CAT2	Catalase isozyme 2
DAMs	Differential accumulation metabolites
DEGs	Differentially expressed genes
NBT	Nitroblue tetrazolium
DAB	Diaminobenzidine
PAL	Phenylalanine ammonia-lyase
POD	Peroxidase
PDA	Potato dextrose agar
PDB	Potato dextrose broth
