# Supplementary material for: The Defense Mechanism of PpCAD4 in Physcomitrium patens Against Botrytis cinerea
Source: Plants (Basel). 2026 Jan 29;15(3):413. doi: 10.3390/plants15030413 (PMC12899312; doi:10.3390/plants15030413)
Supplement: Supplementary file 1 [file plants-15-00413-s001.zip › Supporting Note S1.pdf]

Supporting note S1

1. Analysis of Widely targeted metabolome

1.1 P. patens preparation and extraction

The samples of *P. patens* treated with *Botrytis cinerea* at 0,6, and 12 hpi were collected. Each treatment had three biological replicates. Samples were subjected to vacuum freeze-drying using a lyophilizer (Scientz-100F), followed by grinding into a fine powder with a mixer mill (MM 400, Retsch) at 30 Hz for 1.5 min. A 50 mg aliquot of the powdered sample was accurately weighed using an electronic balance (MS105DM) and mixed with 1,200 μL of pre-chilled 70% methanol aqueous solution containing an internal standard at -20 °C. The mixture was vortexed for 30 sec at 30 min intervals, six times in total. After centrifugation at 15,000 × g for 3 minutes, the supernatant was carefully transferred and filtered through a 0.22 μm microporous membrane before being transferred into injection vials for UPLC-MS/MS analysis.

1.2 UPLC Conditions

The sample extracts were analyzed using an UPLC-ESI-MS/MS system (UPLC, ExionLC™ AD，https://sciex.com.cn/; MS, Applied Biosystems 6500 Q TRAP, https://sciex.com.cn/). The UPLC analytical conditions were as follows: column, Agilent SB-C18 (1.8 μm, 2.1 mm * 100 mm); solvent A, pure water with 0.1% formic acid, solvent B, acetonitrile with 0.1% formic acid. The effluent was alternatively connected to an ESI-triple quadrupole-linear ion trap-MS. Sample measurements were performed with a gradient program that employed the starting conditions of 95% A, 5% B. Within 9 min, a linear gradient to 5% A, 95% B was programmed, and a composition of 5% A, 95% B was kept for 1 min. Subsequently, a composition of 95% A, 5.0% B was adjusted within 1.1 min and kept for 2.9 min. The flow velocity was set as 0.35 mL per minute, and the column oven was set to 40°C. The injection volume was 2 μL.

1.3. ESI-Q TRAP-MS/MS

The ESI source operation parameters were as follows: source temperature 500°C; ion spray voltage (IS) 5500 V (positive ion mode)/-4500 V (negative ion mode); ion source gas I (GSI), gas II (GSII), curtain gas (CUR) were set at 50, 60, and 25 psi, respectively; the collision-activated dissociation (CAD) was high. QQQ scans were acquired as MRM experiments with collision gas (nitrogen) set to medium. DP (declustering potential) and CE (collision energy) for individual MRM transitions was done with

further DP and CE optimization. A specific set of MRM transitions were monitored for each period according to the metabolites eluted within this period.

2. Construction of P. patens cDNA library in yeast

2.1. Extraction of total RNA and construction and detection of cDNA primary library

A total of 0.1 g of healthy and uniformly growing *P. patens* was accurately weighed and ground to a fine powder in liquid nitrogen. Total RNA was extracted using the Trizol method, followed by mRNA isolation and purification. The total RNA extracted from *P. patens* was used as the template to synthesize the first strand cDNA by reverse transcription, and then the synthesized first strand cDNA was used as template to synthesize the second strand cDNA under the action of *E. coli* DNA Ligase (10 U/μL), *E. coli* DNA Polymerase I (10 U/μL), *E. coli* RNaseH (2 U/μL) and T4 DNA Polymerase. The obtained cDNA related to the three-frame attB1 recombinant joint, and the cDNA was collected after fractionation by cDNA. The collected cDNA was recombined with BP and transformed into *E. coli* DH10B cells. After transformation, it was put into SOC medium and cultured in a shaking bed of 300 × g at 37℃ for 1 h. A total of 10 μL bacterial solution was taken to determine the storage capacity, recombination rate and inserted fragment length. The remaining culture was added with glycerol until the final concentration was 20% and stored at -80℃.

2.2 Construction and preservation of a yeast cDNA library

The primary cDNA library plasmid was extracted and diluted to 300 ng/μL. It was subsequently recombined with the destination vector pGADT7-DEST via LR clonase-mediated recombination. The recombinant plasmid was transformed into *E. coli* DH10B competent cells, and the resulting yeast cDNA library was plated on SOC medium. A 10 μL aliquot of the transformed bacteria was used for quality assessment of the library. The remaining bacterial suspension was preserved in 20% glycerol at −80 °C.
