# Supplementary figures and images for: The Defense Mechanism of PpCAD4 in Physcomitrium patens Against Botrytis cinerea
Source: Plants (Basel). 2026 Jan 29;15(3):413. doi: 10.3390/plants15030413 (PMC12899312; doi:10.3390/plants15030413)

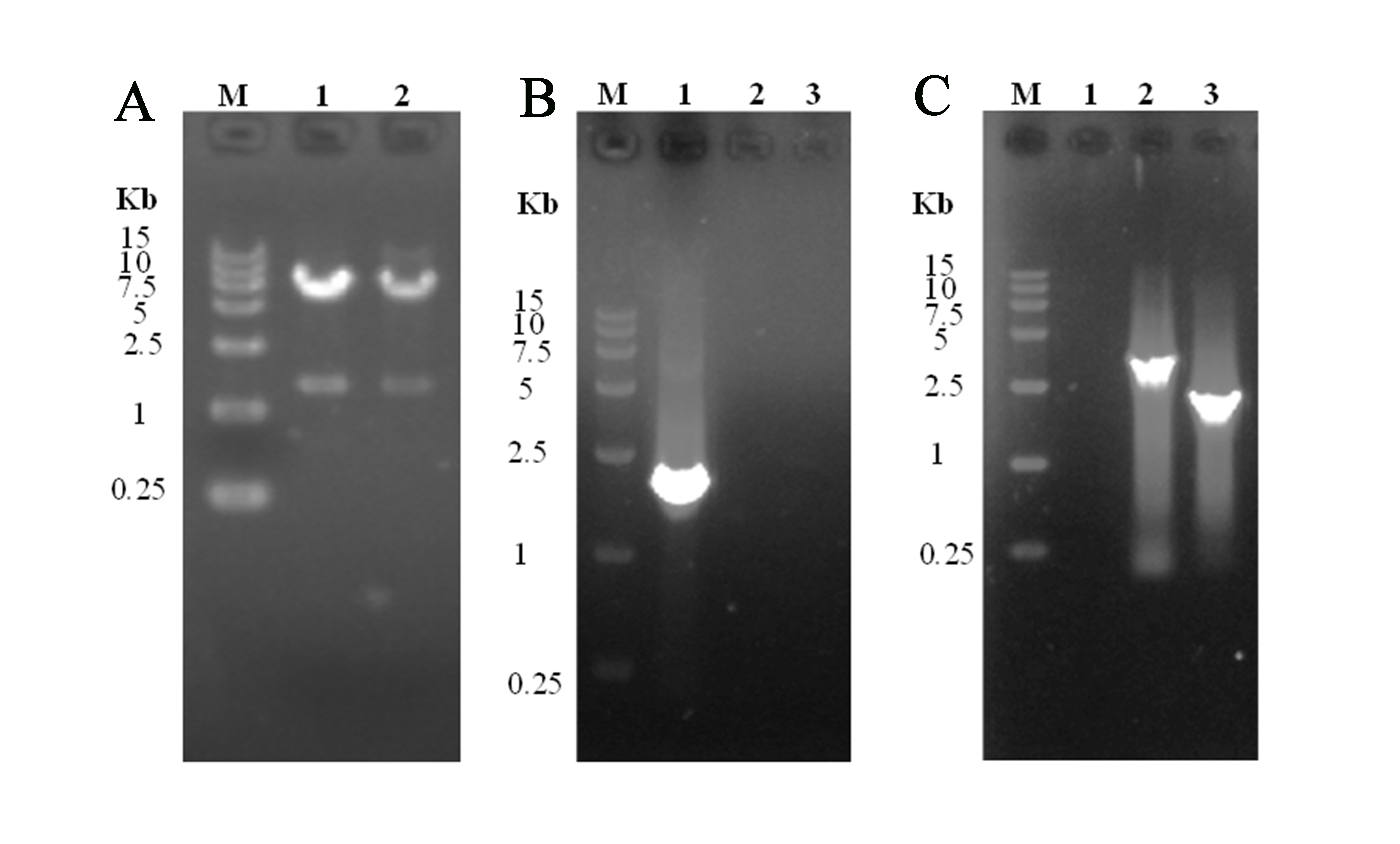

Supplement: Supplementary file 1 [file plants-15-00413-s001.zip › Figure S1.tif]

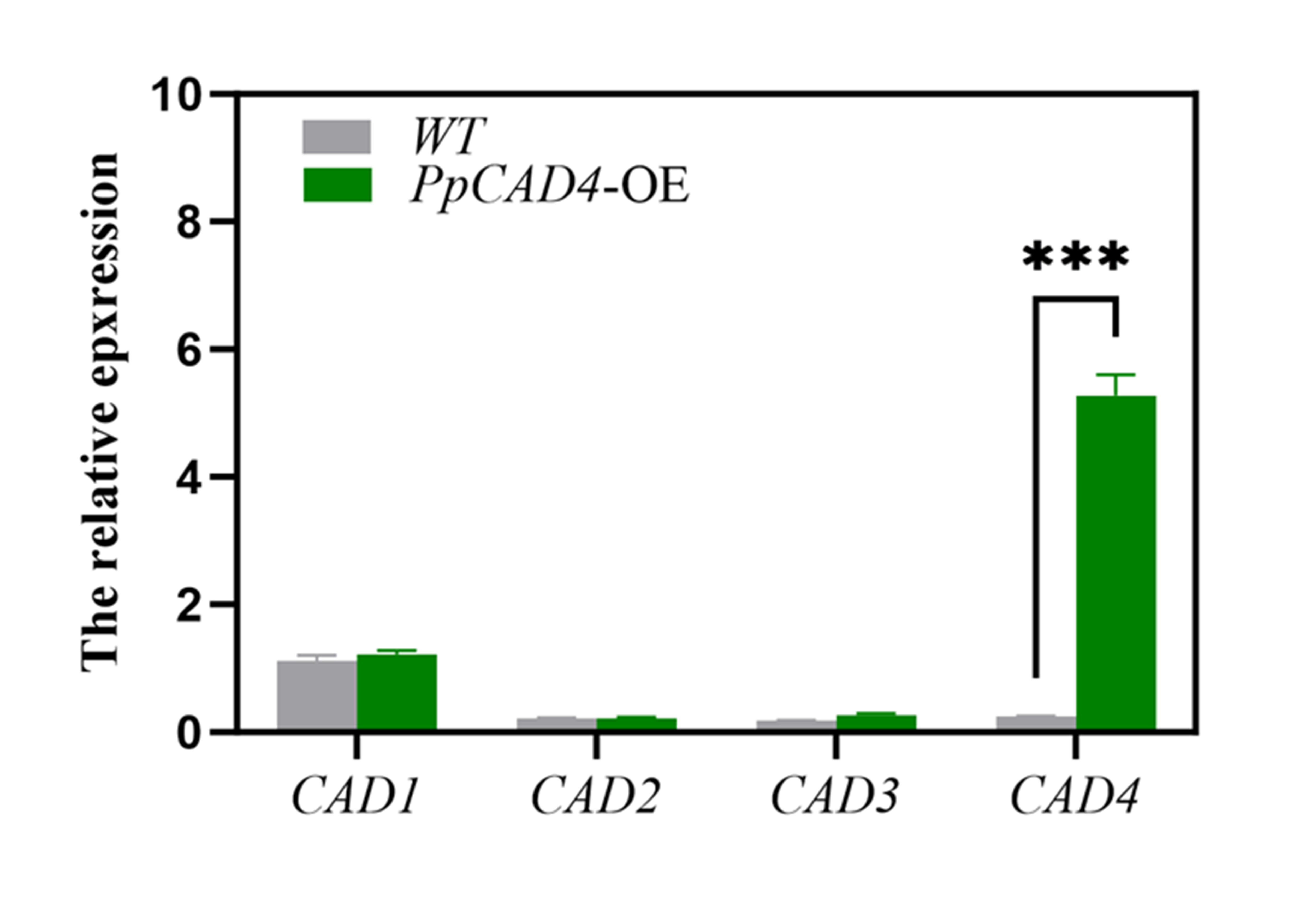

Supplement: Supplementary file 1 [file plants-15-00413-s001.zip › Figure S2.tif]

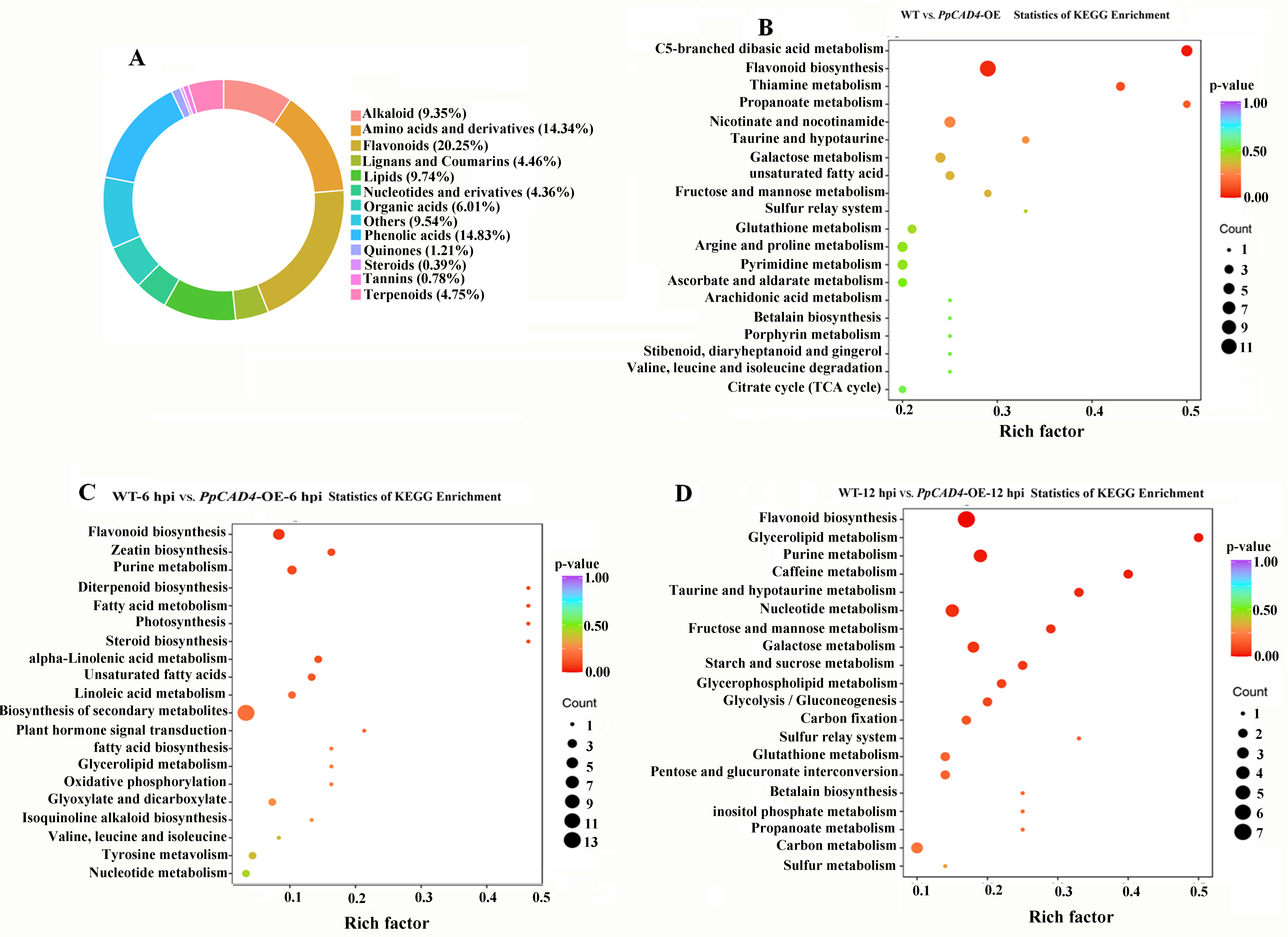

Supplement: Supplementary file 1 [file plants-15-00413-s001.zip › Figure S4.tif]

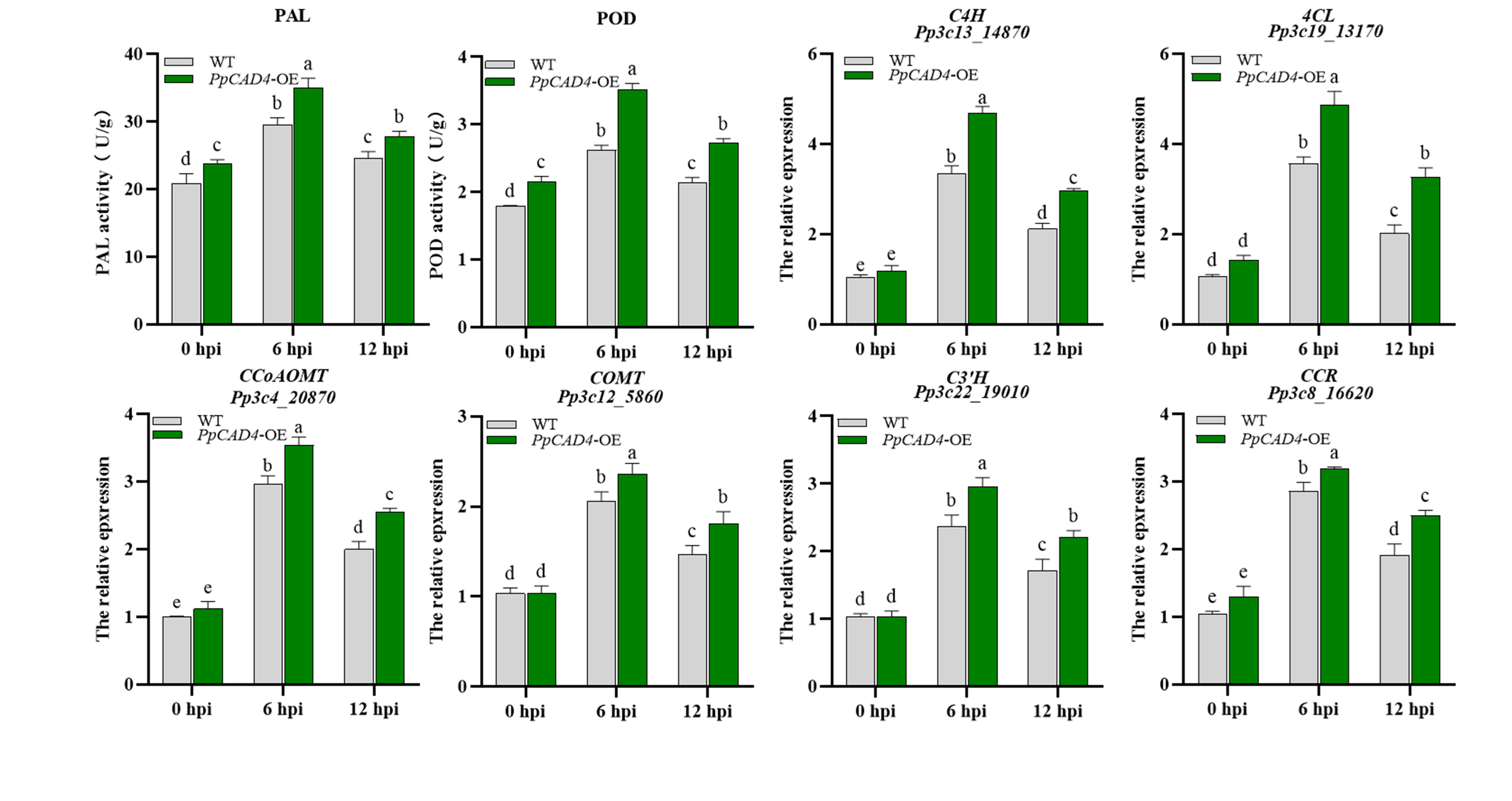

Supplement: Supplementary file 1 [file plants-15-00413-s001.zip › Figure S5.tif]
